# Supplementary material for: Prussian Blue Nanoparticle-Mediated Scalable Thermal Stimulation for In Vitro Neuronal Differentiation
Source: Nanomaterials (Basel). 2022 Jul 4;12(13):2304. doi: 10.3390/nano12132304 (PMC9268683; doi:10.3390/nano12132304)
Supplement: Supplementary file 1 [file nanomaterials-12-02304-s001.zip › nanomaterials-1737401-supplementary.pdf]

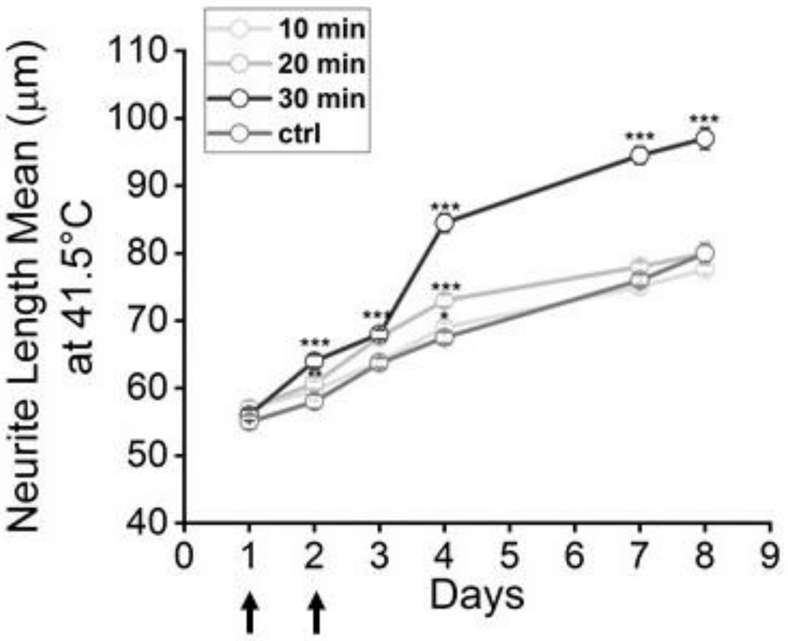

Figure S1. Neurite length of F-11 cells heated (41.5°C) on day 1 and day 2 after seeding (black arrows) at different times of exposure. 10 minutes did not induce any effect on cell morphology compared to the control. Cells maintained at 41.5°C for 20 minutes showed neurites significantly longer versus the control only on day 2 ($p = 0.01$, Mann-Whitney Test) and 4 ($p = 1.15 \times 10^{-6}$, Mann-Whitney Test). Cells maintained at 41.5°C for 30 minutes showed neurites significantly longer compared to the control starting from day 2 ($p < 0.001$, Mann-Whitney Test).

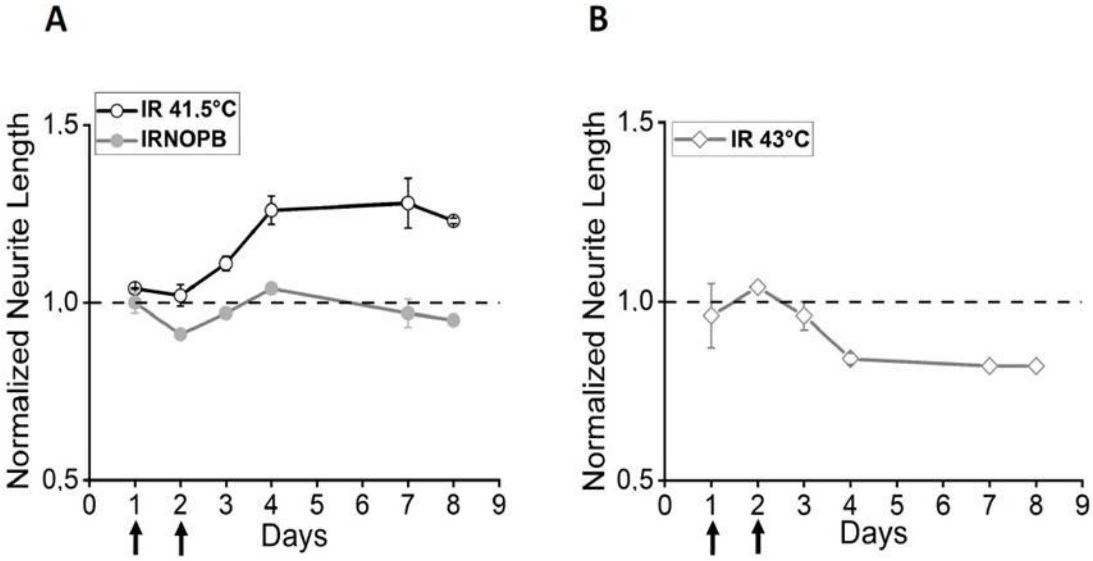

Figure S2. (**A**) Morphological properties of irradiated F-11 cells on day 1 and day 2 after seeding (black arrows) with and without PBNPs. Mean neurite length was longer in irradiated samples with PBNPs compared to both cells irradiated without PBNPs and control. Moreover, cells irradiated without PBNPs showed a trend similar to the control for all the days of the experiment. (**B**) Morphological properties of irradiated F-11 cells at 43°C. Irradiated samples at 43°C showed shorter neurites and seemed to be more stressed compared to the control, which had a neurite length mean significantly longer starting from day 3, as seen in bulk heating experiments.
